# Anisotropic Magnetoresistance of Nano-conductive Filament in Co/HfO_2_/Pt Resistive Switching Memory

**DOI:** 10.1186/s11671-017-1983-2

**Published:** 2017-03-22

**Authors:** Leilei Li, Yang Liu, Jiao Teng, Shibing Long, Qixun Guo, Meiyun Zhang, Yu Wu, Guanghua Yu, Qi Liu, Hangbing Lv, Ming Liu

**Affiliations:** 1 Department of Materials Physics and Chemistry, University of Science and Technology Beijing, Beijing, 100083 China; 2 Key Laboratory of Microelectronic Devices & Integrated Technology, Institute of Microelectronics, Chinese Academy of Sciences, Beijing, 100029 China; 3 Nanoscale Physics & Devices Laboratory, Institute of Physics, Chinese Academy of Sciences, Beijing, 100190 China; 4 University of Chinese Academy of Sciences, Beijing, 100049 China; 5 Jiangsu National Synergetic Innovation Center for Advanced Materials (SICAM), Nanjing, 210023 China

**Keywords:** Conductive bridge random access memory, Resistive switching, Conductive filament, Anisotropic magnetoresistance

## Abstract

Conductive bridge random access memory (CBRAM) has been extensively studied as a next-generation non-volatile memory. The conductive filament (CF) shows rich physical effects such as conductance quantization and magnetic effect. But so far, the study of filaments is not very sufficient. In this work, Co/HfO_2_/Pt CBRAM device with magnetic CF was designed and fabricated. By electrical manipulation with a partial-RESET method, we controlled the size of ferromagnetic metal filament. The resistance-temperature characteristics of the ON-state after various partial-RESET behaviors have been studied. Using two kinds of magnetic measurement methods, we measured the anisotropic magnetoresistance (AMR) of the CF at different temperatures to reflect the magnetic structure characteristics. By rotating the direction of the magnetic field and by sweeping the magnitude, we obtained the spatial direction as well as the easy-axis of the CF. The results indicate that the easy-axis of the CF is along the direction perpendicular to the top electrode plane. The maximum magnetoresistance was found to appear when the angle between the direction of magnetic field and that of the electric current in the CF is about 30°, and this angle varies slightly with temperature, indicating that the current is tilted.

## Background

In recent years, the requirements for non-volatile become more and more strict in the storage density, operation speed, and power consumption [1–7]. Resistive Random Access Memory (RRAM), which has the advantages of high integration, low power consumption, high read-write speed, and compatibility with CMOS technology, is regarded as one of the most promising new memories [8–10]. RRAM is based on the reversible switching between high and low resistance states (HRS and LRS) of a metal/insulator/metal structure in response to the external electric field [11, 12]. According to the difference of the electrode and switching mechanism, the metal-oxide RRAM can be divided into electrochemical metallization memory (ECM) and valence change memory (VCM) [13, 14]. The ECM device is also called conductive bridge random access memory (CBRAM). In CBRAM, the SET and RESET switchings are attributed to the connection and rupture of metallic conducting filaments (CFs) in metal oxides, respectively. The CFs are formed by the metal ions transferring from the top electrode to the bottom electrode through the electrochemical metallization effect [13–15]. In previous studies, researchers have focused on the electrically manipulated conductive filament. If the magnetic structures can be manipulated at the same time, it is possible to construct multilevel memory devices for exploration. RRAM is an excellent system for controlling both electrical and magnetic properties at the same time. Therefore, the electrical manipulation of magnetic properties in RRAM is a hot issue.

Recently, the Ni CF in RRAM has been confirmed to show ferromagnetism [16, 17]. To manipulate ferromagnetism, it is necessary to understand the magnetic structure. As different metal electrodes have obvious influence on the resistive switching behaviors [18], the magnetic structure characteristics in the RRAM devices with different magnetic electrodes such as Fe, Co, or Fe might be different. On the other hand, direct characterization of the magnetic structure is very difficult. Actually, anisotropic magnetoresistance (AMR) [19, 20] is a good method not only to confirm the ferromagnetism but also to give detailed magnetic structure information. HfO_2_ is a widely used resistive switching dielectric which has been demonstrated to show excellent memory performances [21, 22]. Co conductive filament has been proved to be formed in the oxide-based RRAM device using Co as an active metal electrode [23]. Therefore, in this study, we fabricated a kind of CBRAM device with Co/HfO_2_/Pt structure to investigate the magnetic structure characteristics of the filament in the device. We measured the AMR by rotating the direction of the magnetic field and by sweeping the magnitude, respectively. In this way, we obtained the spatial direction as well as the easy-axis of the CF.

## Methods

**Fig. 1:**
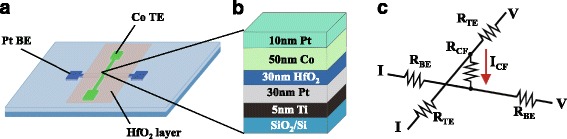
(*Color online*) Schematics of **a** the crossbar RS device structure and **b** the cross section of the device. **c** The schematic of resistance network of the *top* electrode (R_TE_), the *bottom* electrode (R_BE_), and the CF (R_CF_)

The fabricated device has a simple crossbar structure formed on a thermally oxidized Si substrate, as shown in Fig. 1a. The size of cross section is from 5 × 5 to 30 × 30 μm^2^. Figure 1b illustrates the cross section of the Co/HfO_2_/Pt structure. First, sequential Ti/Pt layers (5/30 nm) were deposited by direct current (DC) magnetron sputtering as adhesion layer and bottom electrode. Then, a HfO_2_ (30 nm) layer was deposited on the Pt electrode by radio frequency (RF) magnetron sputtering. Finally, a Co layer (50 nm) was deposited by DC magnetron sputtering as the top electrode and a Pt layer (10 nm) was covered on Co layer to prevent oxidation of Co. The electrical measurements are performed by Keithley 4200-SCS Semiconductor Characterization System at 300 K in atmosphere with DC voltage sweeping mode. The bias polarity is defined with reference to the bottom Pt electrode. In the forming and SET switching, a current compliance of 0.1 mA is limited to prevent the complete dielectric breakdown. By using the Physical Property Measurement System (PPMS), AMR of CF in LRS was measured at 300, 200, 100, and 10 K by rotating the direction of the magnetic field and by sweeping the magnitude, respectively. Figure 1c shows the schematic of resistance network of the device and the electrodes. A four-probe method was used during the measurements of the LRS state and AMR. In the HRS, a two-probe method was used during measurements because resistance of the HRS is much greater than that of electrodes.

## Results and Discussion

**Fig. 2:**
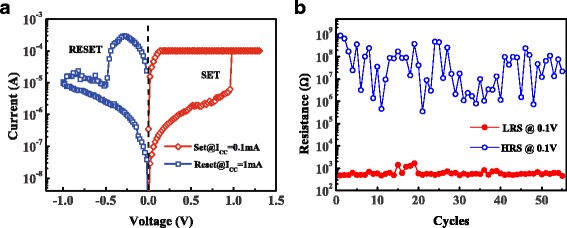
(*Color online*) **a** Typical bipolar RS characteristics. The current compliance value for the SET process is 100 μA. **b** Endurance characteristics under DC sweeping mode. The readout voltage is 0.1 V

The device was operated under positive SET and negative RESET operation mode, suggesting the bipolar switching mechanism. Figure 2a shows the typical bipolar RS characteristic of the Co/HfO_2_/Pt devices. As the positive bias is applied to the Co electrode, the current increases abruptly at ~1 V, which indicates that a transition from HRS to LRS occurs, corresponding to the SET process. When the bias with the opposite polarity is applied, the current decreases abruptly to a low value, which corresponds to the RESET process. The device switches reversibly between HRS and LRS under the DC voltages in alternative polarities. The conductive filament model accounts for the switching mechanism of the device. Figure 2b shows the endurance of RS behavior in DC voltage sweeping mode, where the read voltage is 0.1 V. RS phenomena with a high HRS/LRS ratio can be observed.

**Fig. 3:**
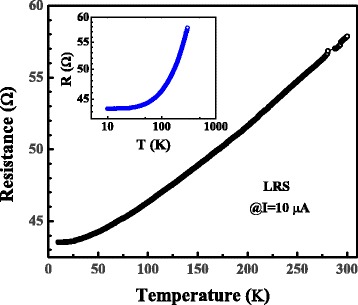
(*Color online*) Temperature dependence of resistance at a constant current of 10 μA in a linear plot. *Inset* is the corresponding semi-logarithmic plot

Figure 3 shows the resistance dependent temperature of the conductive filament in LRS. The resistance of the LRS decreases almost linearly as the temperature is lowered from 280 to 50 K, indicating that the CF has a metallic conduction property. The residual resistivity ratio (RRR), which can be expressed as RRR = R_300 K_/R_10 K_ is 1.33. This value is much smaller than that of the bulk pure Co. It is suggested that the conductive electrons in the CF are scattered by lattice imperfections such as surfaces, grain boundaries, and impurities [24, 25].

**Fig. 4:**
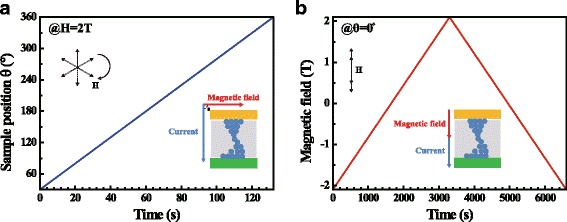
(*Color online*) Two methods for scanning the magnetic field in AMR measurement. **a** scanning angle. **b** scanning magnitude

From the above results, it has been found that the ferromagnetic conductive filament has been formed in the RRAM, but the magnetic structure of the filaments is unclear. To examine the magnetic characteristics of CF, we measured the AMR in two methods as shown in Fig. 4. The first measurement method is fixing the magnitude of the magnetic field at 2 T, and rotating its direction continuously from 0° to 360° (Fig. 4a). The second method is fixing the direction of the magnetic field and scanning its magnitude between +2 T and −2 T. The direction is fixed as θ = 0° as shown in Fig. 4b, i.e., the magnetic field is perpendicular to the substrate and the Co top electrode planes.

**Fig. 5:**
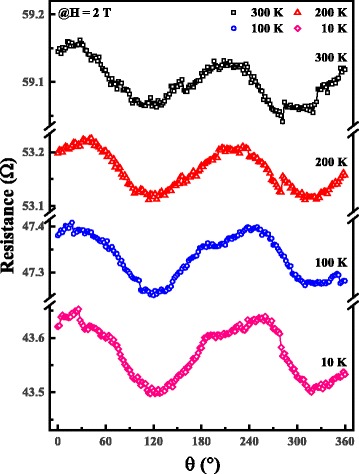
(*Color online*) The magnetoresistance varying as a function of sample angle at different temperatures. The direction and magnitude of the magnetic field are the same as those in Fig. 4a. The inflow of constant current is 10 μA to read the resistance

AMR is given by

1 $$ \rho \left(\theta \right)={\rho}_{{}_{\perp }}{ \sin}^2\theta +{\rho}_{\parallel }{ \cos}^2\theta $$

where θ is the angle between the current in the material, and the direction of the magnetization, ρ_⊥_ and ρ_∥_ are the electrical resistivities perpendicular and parallel to the direction of magnetization, respectively. In the case of ferromagnetic transition metals and alloys, ρ_∥_ is the maximum of the ρ(θ), and ρ_⊥_ is the minimum of the ρ(θ). Figure 5 shows the resistance as a function of the angle between the current and the magnetic field directions at different temperatures. As can be seen from the black curve in Fig. 5, ρ_∥_ appears at about 30° and ρ_⊥_ presents at about 120°. The results indicate that when the direction of electric current in the CF is tilted by about 30° from the magnetic field direction, the magnetoresistance is the maximum. In the measurement of as shown in Fig. 5, the applied magnetic field is large enough and greater than the saturation field, indicating that the magnetization direction tends to be parallel to the direction of the applied magnetic field. When the maximum of ρ appears, the magnetization direction is parallel to the electric current direction. Meanwhile, the angle between the *z* axis and the magnetic field direction is 30°, which means the angle between the *z* axis and the magnetization direction is 30°. Therefore, the angle between the *z* axis and the current direction is 30°.

**Fig. 6:**
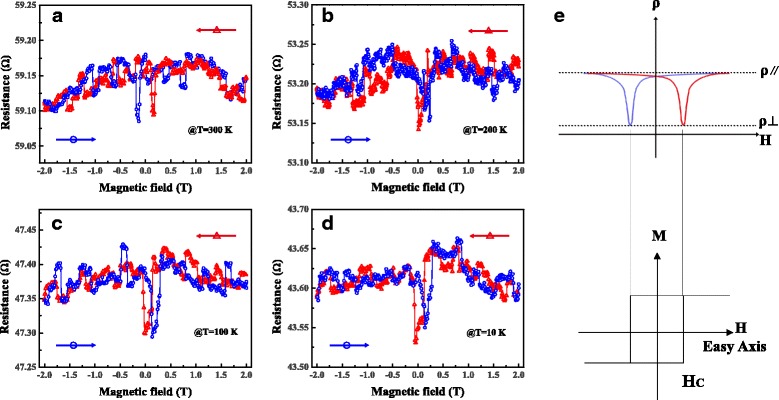
(*Color online*) **a**–**d** Characteristics of magnetoresistance at θ = 0° at different temperatures in LRS. Direction of the external magnetic field is as shown in Fig. 4a. A constant current of 10 μA is applied to read the resistance. The *red triangular line* was tested with the magnetic field swept from + 2 T to −2 T, and the *green circle line* was obtained by sweeping the magnetic field from −2 T to + 2 T. The *arrows* in the figures represent the respective sweep directions. **e** Typical AMR curves and hysteresis loop [32]

Concave AMR curves were observed under the sweeping magnetic field with θ = 0° at different temperatures, as shown in Fig. 6. When the magnetic field sweeps forth and back, the magnetoresistance curves have two different concave peaks at each fixed temperature, indicating that the easy-axis of the CF is along with the direction of the external magnetic field perpendicular to the top electrode plane. As mentioned above, the maximum of ρ in Fig. 5 appears when the magnetization direction is parallel to the electric current direction, which corresponds to ρ_//_. The maximum of ρ in Fig. 6a)appearing at *H* = 0 is close to ρ_//_. It means that the direction of magnetization at zero field is approximately parallel to the direction of electric current, indicating the out-of-plane magnetic anisotropy of the CF. The downward peaks correspond to the position of coercivity, as shown in Fig. 6e.

The fabricated device with ferromagnetic CF exhibits both electric field-induced resistive switching and magnetic field-induced magnetoresistance properties. Although the observed magnitude of AMR is small, there is the possibility of obtaining a large MR, such as using a transistor to achieve a stable current limit to precisely control the size of the magnetic conductive filament. When the size of the magnetic channel is less than the average free path of electrons, ballistic transport will occur, thus large MR effect might be obtained [26–30]. Furthermore, in the low-temperature environment, the quantum effect will become more significant [31].We expect that the ferromagnetic CF will pave the way to a new multi-functional device with both spin-dependent and electrical field-dependent conduction properties, not only for memory but also for logic device functions as well.

## Conclusions

We investigated the resistive switching characteristics of the ferromagnetic CF in RRAM device with a Co/HfO_2_/Pt structure. Bipolar switching was observed. The temperature dependence of the resistance of the CF formed in the device exhibits metallic conduction properties. It was shown that AMR occurs in the LRS, which strongly suggests that a ferromagnetic CF is formed in the HfO_2_ layer. Through analyzing the AMR phenomenon produced by the conductive filament, the maximum magnetoresistance appears when angle between the direction of magnetic field and that of the electric current in the CF is about 30°, while the easy-axis of the CF is along the direction perpendicular to the top electrode plane.

## Abbreviations

AMR: Anisotropic magnetoresistance
CBRAM: Conductive bridge random access memory
CF: Conductive filament
ECM: Electrochemical metallization memory
HRS: High resistance states
LRS: Low resistance states
PPMS: Physical property measurement system
RRAM: Resistive random access memory
RS: Resistive switching
